# Genetic associations between circulating metabolic biomarkers and lung cancer in East Asians and Europeans

**DOI:** 10.1186/s40001-023-01116-4

**Published:** 2023-04-26

**Authors:** Kai Liu, Shangshang Wang, Yuhan Zhou, Sha Huang, Yifan Liu, Lijiang Song, Zhengfu He

**Affiliations:** 1 Department of Thoracic Surgery, Sir Run Run Shaw Hospital School of Medicine, Zhejiang University, Hangzhou, Zhejiang China; 2 Nursing department, Sir Run Run Shaw Hospital, School of Medicine, Zhejiang University, Hangzhou, Zhejiang China

**Keywords:** Metabolic biomarkers, LDL, Triglyceride, Mendelian randomization, Lung cancer

## Abstract

**Background:**

Metabolic biomarkers are reported to be associated with the risk of lung cancer (LC). However, the observed associations from epidemiological studies are either inconsistent or inconclusive.

**Methods:**

The genetic summary data of high-density lipoprotein cholesterol (HDL), low-density lipoprotein cholesterol (LDL), total cholesterol (TC), triglyceride (TG), fasting plasma glucose (FPG), and glycated hemoglobin (HbA1c) and those of the LC and its histological subtypes were retrieved from previous GWASs. We performed two-sample Mendelian randomization (MR) and multivariable MR analyses to examine the associations between genetically predicted metabolic biomarkers and LC in East Asians and Europeans.

**Results:**

In East Asians, the inverse-variance weighted (IVW) method suggests that LDL (odds ratio [OR] = 0.799, 95% CI 0.712–0.897), TC (OR = 0.713, 95% CI 0.638–0.797), and TG (OR = 0.702, 95% CI 0.613–0.804) were significantly associated with LC after correction for multiple testing. For the remaining three biomarkers, we did not detect significant association with LC by any MR method. Multivariable MR (MVMR) analysis yielded an OR of 0.958 (95% CI 0.748–1.172) for HDL, 0.839 (95% CI 0.738–0.931) for LDL, 0.942 (95% CI 0.742–1.133) for TC, 1.161 (95% CI 1.070–1.252) for TG, 1.079 (95% CI 0.851–1.219) for FPG, and 1.101 (95% CI 0.922–1.191) for HbA1c. In Europeans, the univariate MR analyses did not detect significant association between exposures and outcomes. However, in MVMR analysis integrating circulating lipids and lifestyle risk factors (smoking, alcohol drinking, and body mass index), we found that TG was positively associated with LC in Europeans (OR = 1.660, 95% CI 1.060–2.260). Subgroup and sensitivity analysis yielded similar results to the main analyses.

**Conclusions:**

Our study provides genetic evidence that circulating levels of LDL was negatively associated with LC in East Asians, whereas TG was positively associated with LC in both populations.

**Supplementary Information:**

The online version contains supplementary material available at 10.1186/s40001-023-01116-4.

## Introduction

In 2020, lung cancer (LC) is the second most commonly diagnosed malignancy and the leading cause of cancer death worldwide, representing approximately 2.2 million cancers diagnosed and 1.8 million deaths [1]. LC are categorized as small cell carcinoma (SCLC) and non-small cell carcinoma; the latter mainly represents by adenocarcinoma (LUAD) and squamous cell carcinoma (LUSC). Worldwide, more than 60% of LC-related deaths are attributable to smoking; others are caused by occupational workplace exposure, air pollution, and diet [2]. Of note is that metabolic factors, such as high plasma glucose level and type 2 diabetes, are also associated with both LC development and prognosis [2–6], indicating that metabolic factors should not be underestimated or even neglected in the prevention and management of LC. On the other hand, the prevalence of metabolic syndrome was rapidly increased over the last decades [7, 8], suggesting a persistent increase of metabolic-related LC burden in the future.

Previous epidemiological studies have examined the association between metabolic factors and the risk of LC. For example, individuals with impaired fasting glucose or diabetes mellitus, or dyslipidemia had an increased risk of LC [9–11]. However, studies reporting a null association remained [12–15], indicating further investigations are warranted. The inconsistency may be attributed to the between-study heterogeneities and the inherent pitfalls of observational studies, including under-adjustment for confounders, small sample size, and reverse causality.

The accumulation of genetic information from genome-wide association study (GWAS) and the advent of genetic methods, such as Mendelian randomization (MR) analysis, provide us the opportunity to further understand the correlation between exposure and outcome. The MR estimates reveal the genetic association between exposure and outcome, which can be viewed as causation if all of the MR assumptions were met [16]. There were several MR analyses have been performed for LC [17–19]. However, the genetic associations between metabolic factors and LC have not been thoroughly studied in populations with different ethnicities. To fill this gap, we used GWAS summary data to examine the genetic associations of six metabolic biomarkers with LC and its histological subtypes in East Asians and Europeans.

## Methods

### GWAS of exposures

In this study, we included six circulating metabolic biomarkers, including high-density lipoprotein cholesterol (HDL), low-density lipoprotein cholesterol (LDL), total cholesterol (TC), triglyceride (TG), fasting plasma glucose (FPG), and glycated hemoglobin (HbA1c), as the exposures. The GWAS summary data of the six biomarkers of East Asians were retrieved from Korea Biobank Array Project [20], in which 125,872 Koreans were involved and 8.3 million variants were genotyped and imputed (Table 1). For Europeans, the GWAS of blood lipid traits and glucose traits were retrieved from the Global Lipids Genetics Consortium (GLGC) [21] and the MAGIC (the Meta-Analyses of Glucose and Insulin-related traits Consortium) [22, 23], respectively (Table 1). Of note is that the GWAS performed by the GLGC and the MAGIC was meta-analysis that aggregated GWAS results from individuals with different genetic ancestry groups. In this study, we only retrieved the GWAS summary data of European people. The details of quality control and statistical analysis of the exposure to GWAS have been shown in previous studies [20–23].

**Table 1:** GWAS sources for lung cancer and metabolic biomarkers in Europeans and East Asians

	Europeans				East Asians			
	PMID	Sample size	Case number	Web source	PMID	Sample size	Case number	Web source
Exposures								
HDL	34887591	1,320,016	–	Global Lipids Genetics Consortium (http://csg.sph.umich.edu/willer/public/glgc-lipids2021/)	36333282	125,872	–	Korea Biobank Array Project (https://www.koreanchip.org/kba130k)
LDL								
TC								
Triglyceride								
FPG	33402679	151,188	–	MAGIC (https://magicinvestigators.org/downloads/)				
HbA1c	28898252	123,655						
Smoking^a^	30643258	518,633	–	GWAS-Catalog: GCST007327	NA	NA	NA	NA
Alcohol drinking^b^	30643258	414,343		GWAS-Catalog: GCST007328	NA	NA	NA	NA
Body mass index	30124842	381,275		IEU OpenGWAS project: ieu-b-40 (https://gwas.mrcieu.ac.uk/datasets/ieu-b-40/)	NA	NA	NA	NA
Outcomes								
Lung cancer	28604730	85,716	29,266	GWAS-Catalog: GCST004748	34594039	178,726	4444	BioBank Japan PheWeb (https://pheweb.jp/pheno/LuC)
LUAD		66,756	11,273	GWAS-Catalog: GCST004744	NA	NA	NA	NA
LUSC		63,053	7426	GWAS-Catalog: GCST004750	NA	NA	NA	NA
SCLC		24,108	2664	GWAS-Catalog: GCST004746	NA	NA	NA	NA

*FPG* fasting plasma glucose, *HbA1c* glycosylated hemoglobin, *HDL* high-density lipoprotein cholesterol, *LDL* low-density lipoprotein cholesterol, *TC* total cholesterol, *SCLC* small cell lung carcinoma, *LUAD* lung adenocarcinoma, *LUSC* lung squamous cell carcinoma, *NA* not available

^a^smoking status (ever vs. never smokers)

^b^Alcohol consumption (drinks per week)

### GWAS of outcomes

LC and its histological subtypes SCLC, LUAD, and LCSC were the outcomes. To avert the bias of the winner’s curse, we selected outcomes from the populations that are independent with that of the exposures. For East Asians, we retrieved the GWAS data of LC from the BioBank Japan (BBJ) that involved 174,282 controls and 4444 LC cases (Table 1) [24]. The LC cases were identified from the electronic medical records. However, the histological subtypes of LC were not available in the BBJ. For Europeans, we retrieved the GWAS data of LC and its subtypes from the McKay JD et al. study [25]. In the McKay study, 85,716 participants were included, among which 29,266 were LC cases (11,273 LUAD; 7426 LUSC; and 2664 SCLC; Table 1), and four independent GWASs were performed for LC and its three subtypes. This is the largest GWAS for lung cancer to date performed in Europeans.

### Genetic instrumental variables

We performed a suite of quality control processes to select eligible genetic instrumental variables (IVs) from the GWAS of exposures. First, we extracted the SNPs that reached significance at the genome-wide level (i.e., *P* < 5 × 10^–8^). Second, we clumped the SNPs to avert linkage disequilibrium (LD) using a reference genome panel (*R*^2^ < 0.01, window size = 10,000 kb). In this process, we retained the SNPs that had a lower *P* value among those pairs of SNPs that had LD *R*^2^ above the specified threshold (0.01). Third, we removed SNPs with a minor allele frequency (MAF) < 1%. Fourth, we matched and extracted the selected SNPs from the GWAS of outcomes. For SNPs that are absent in the outcome GWAS, we retrieved a proxy SNP that had an LD *R*^2^ > 0.8 with the requested SNP. Finally, ambiguous SNPs with unconcordant alleles and palindromic SNPs with an ambiguous strand were either directly excluded or corrected in MR analysis.

To test whether the selected instrumental SNPs are strongly associated with exposure (the first assumption of MR analysis [26]), we calculated the F-statistic using the following formula: *F* = *R*^*2*^*(n − k − 1)/k(1 − R*^*2*^*)*, where *R*^*2*^, *n*, and *k* denote the proportion of variance of exposure explained by selected genetic tools, a sample size of exposure GWAS, and a number of selected IVs, respectively [27]. *R*^2^ can be calculated by *2* × *β*^*2*^ × *EAF* × *(1-EAF)*, where β is the estimated coefficient of the IV in exposure GWAS and EAF is the effect allele frequency [28]. A mean F-statistic > 10 suggests suitable IVs [29].

### Mendelian randomization analysis

The methodological details of two-sample MR analysis have been presented elsewhere [30]. In this study, we used the inverse-variance weighted (IVW) method to examine the genetic associations between exposures and outcomes. The IVW method uses a meta-analysis approach to combine Wald estimates for each SNP (i.e., the β coefficient of the SNP for exposure divides by the β coefficient of the SNP for outcome) to get the overall estimates of the effect [31]. We also applied the IVW method to test between-SNP heterogeneity. *The p* value of the Q-statistic > 0.05 means the absence of heterogeneity. We used the MR-Egger regression intercept test to identify the horizontal pleiotropy. Since it is unlikely that all genetic variants would be valid IVs, several robust methods, including weighted-median, weighted-mode, and MR-Egger regression methods, were used as sensitivity analyses. To avoid the impact of outliers, we used the MRPRESSO method to correct the MR estimates by excluding potential outliers [32]. We also performed multivariable MR (MVMR) analysis to adjust for potential pleiotropy. In this analysis, we included all of the six metabolic biomarkers in the MR analysis and the IVs were the combinations of the IV of each exposure. We also performed a MVMR analysis that further incorporated lifestyle risk factors for LC (i.e., smoking, body mass index [BMI], and alcohol drinking) if the GWAS summary data were available [33]. The GWAS summary data of smoking, BMI, and alcohol drinking were retrieved from respective GWAS (Table 1). Of note is that the MVMR analysis including both serum lipids and lifestyle risk factors was only performed for Europeans because the GWAS summary data of smoking, alcohol drinking, and BMI were not available for East Asians. All statistical analyses were performed using the R program (v4.0.3). We used false discovery rate (FDR) to adjust for multiple testing and an FDR < 0.05 was deemed statistically significant.

## Results

### Association between metabolic biomarkers and lung cancer in East Asians

**Fig. 1:**
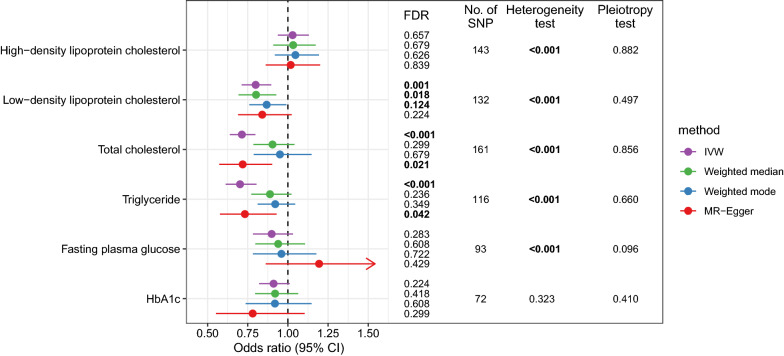
The associations between metabolic biomarkers and lung cancer in East Asians according to Mendelian randomization analysis. (*HbA1c* glycated hemoglobin, *FDR* false discovery rate, *IVW* inverse-variance weighted; Numbers in bold denote statistically significant.)

**Fig. 2:**
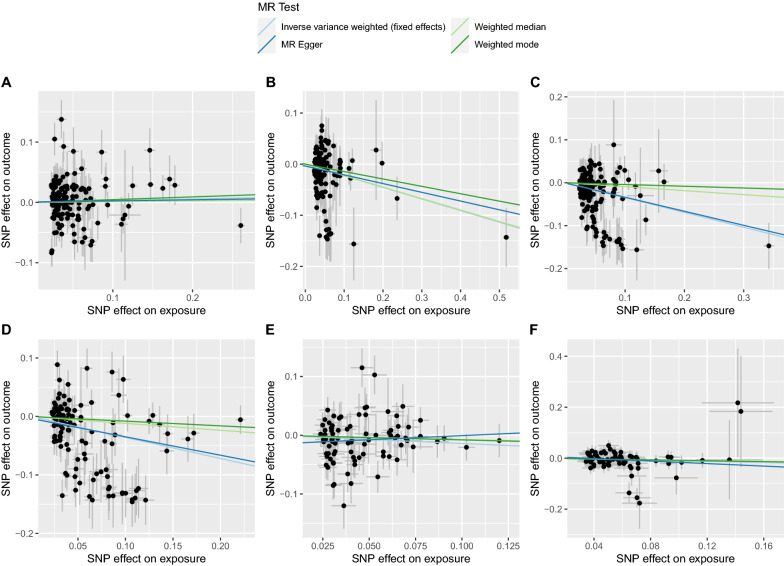
The scatter plots showing SNP effects on both exposures and outcomes in East Asians. (**A**, high-density lipoprotein cholesterol; **B**, low-density lipoprotein cholesterol; **C**, total cholesterol; **D**, triglyceride; **E**, fasting plasma glucose; **F**, glycated hemoglobin.)

In this analysis, there were 72 to 161 SNPs that were used as IVs for exposures (Additional file 1: Tables S1–S6). The mean F-statistics for every instrument-exposure association were > 10 in our study (from 37.5 to 149.8), suggesting a small possibility of weak instrumental variable bias. The MR estimates from different methods of assessing the genetic association of six metabolic factors with LC in East Asians are presented in Fig. 1 and Additional file 1: Table S7. The IVW method suggests that LDL (odds ratio [OR] = 0.799, 95% CI 0.712–0.897), TC (OR = 0.713, 95% CI 0.638–0.797), and TG (OR = 0.702, 95% CI 0.613–0.804) were significantly associated with LC after correction for multiple testing (Fig. 1). The estimates of other three MR methods were similar to that of the IVW method, although most of them were statistically non-significant. For the remaining three biomarkers, HDL, FPG, and HbA1c, we did not detect a significant association with LC by any MR method. The scatter plots showing SNP effects on both exposure and outcome are shown in Fig. 2. In these analyses, we detected significant between-SNP heterogeneity for all biomarkers except for HbA1c. However, no horizontal pleiotropy was found for any biomarker.

### Association between metabolic biomarkers and lung cancer in Europeans

**Fig. 3:**
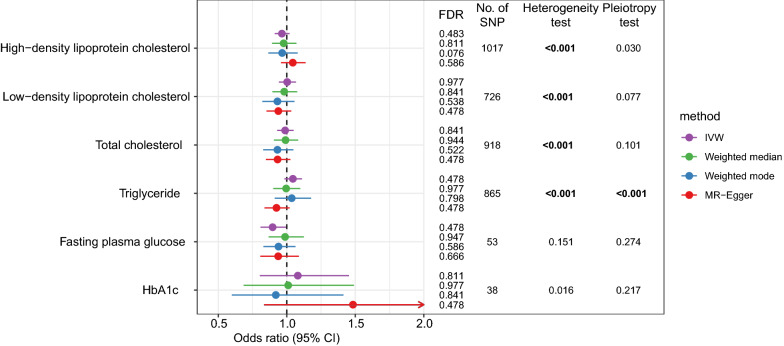
The associations between metabolic biomarkers and lung cancer in Europeans according to Mendelian randomization analysis. (*HbA1c* glycated hemoglobin, *FDR* false discovery rate, *IVW* inverse-variance weighted; Numbers in bold denote statistically significant.)

**Fig. 4:**
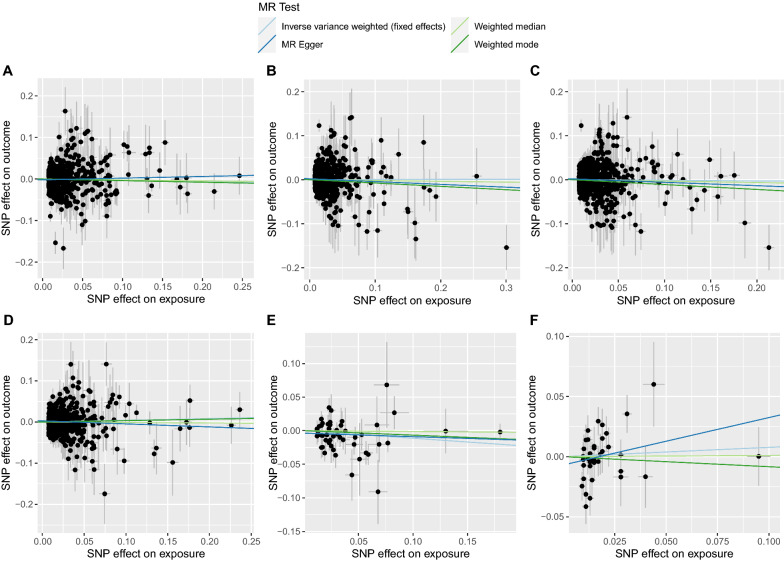
The scatter plots showing SNP effects on both exposures and outcomes in Europeans. (**A**, high-density lipoprotein cholesterol; **B**, low-density lipoprotein cholesterol; **C**, total cholesterol; **D**, triglyceride; **E**, fasting plasma glucose; **F**, glycated hemoglobin.)

There were 38 to 1017 SNPs that were used as IVs for exposures in this analysis (Additional file 1: Tables S8–S13). The mean F-statistics for every instrument-exposure association were much greater than 10 (from 109.5 to 578.9) in this analysis. The IVW method found between-SNP heterogeneity for most exposures, whereas the horizontal pleiotropy was only detected for TG (Fig. 3). The IVW method suggests no significant association between metabolic biomarkers and LC; the OR was 0.964 (95% CI 0.912–1.020) for HDL, 1.003 (95% CI 0.942–1.067) for LDL, 0.988 (95% CI 0.930–1.049) for TC, 1.044 (95% CI 0.982–1.111) for TG, 0.896 (95% CI 0.808–0.995, *FDR* = 0.478), and 1.080 (95% CI 0.803–1.454) for HbA1c (Fig. 3; Additional file 1: Table S14). No conflicting result was found by other MR methods. The scatter plots showing SNP effects on both exposure and outcome are shown in Fig. 4.

We also examined the associations between metabolic biomarkers and LUAD, LUSC, and SCLC. The results are shown in Additional file 2: Figs. S1–S6 and Additional file 1: Table S14. We did not detect any significant association in these analyses. Different MR methods gave similar estimates. MRPRESSO method also yielded similar results to the main analysis when excluding outliers (Additional file 2: Fig. S7).

### MVMR

**Fig. 5:**
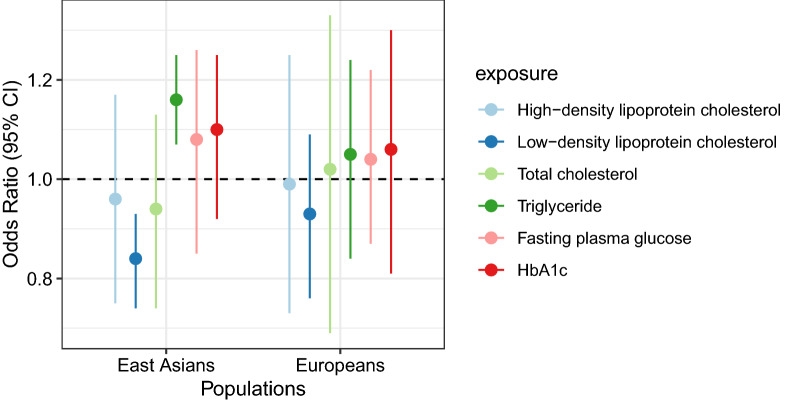
The associations between metabolic biomarkers and lung cancer in East Asians and Europeans according to multivariable Mendelian randomization analysis

**Fig. 6:**
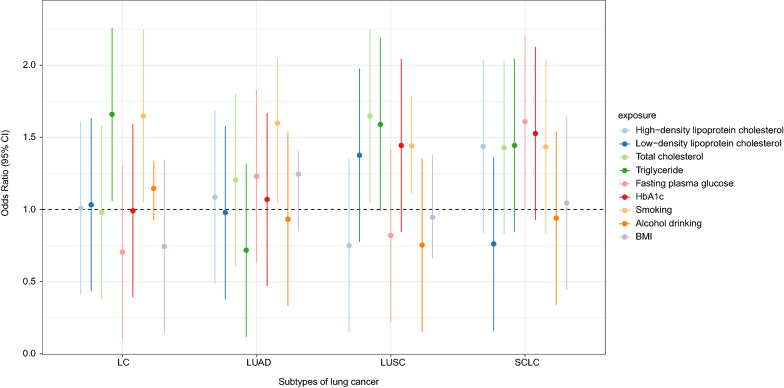
The association between metabolic biomarkers and lifestyle risk factors and lung cancer in Europeans according to multivariable Mendelian randomization analysis

Figure 5 displays the estimates of MVMR in East Asians and Europeans. In East Asians, 478 SNPs were used as the IVs and the MVMR yielded an OR of 0.958 (95% CI 0.748–1.172) for HDL, 0.839 (95% CI 0.738–0.931) for LDL, 0.942 (95% CI 0.742–1.133) for TC, 1.161 (95% CI 1.070–1.252) for TG, 1.079 (95% CI 0.851–1.219) for FPG, and 1.101 (95% CI 0.922–1.191) for HbA1c. In Europeans, 2649 SNPs were used as the IVs and we found that metabolic biomarkers were not significantly associated with LC in this population. For LC subtypes LUAD, LUSC, and SCLC, we did not detect a significant association between exposure and outcome according to the MVMR method either (Additional file 2: Fig. S8). When further adjusted for the effect of smoking, alcohol drinking, and BMI, we found that TG was positively associated with LC in Europeans (OR = 1.660, 95% CI 1.060–2.260) (Fig. 6; Additional file 1: Table S15).

## Discussion

In this study, we used GWAS summary data of metabolic biomarkers and lung cancer to examine the genetic association between these two traits. In East Asians, the IVW method suggests that LDL, TC, and TG were inversely associated with LC. MVMR analysis reports a negative association of LDL and a positive association of TG, respectively, with LC. However, the negative association between LDL and LC was not replicated by either univariate or multivariable MR analysis in Europeans. Subgroup and sensitivity analyses gave similar results to the main analysis.

Blood lipids have long been reported to involve in LC development. However, evidence from epidemiological studies was scattered and inconclusive [34, 35]. A meta-analysis showed a significant inverse association between HDL, TC, and the risk of LC, whereas reported a significantly positive association between serum TG levels and the LC risk [35]. Chandler et al. using data from Women’s Health Study reported that HDL was negatively associated with LC risk, but a significant association was not detected for either TC or TG [36]. In a Chinese population, Lyu et al. found that low levels of LDL were significantly associated with an increased risk of LC, whereas in subjects having high levels of serum LDL, the risk of LC was comparable with that of the reference group [37]. No significant association was detected between HDL, TG, and LC in this study [37]. Compared to these epidemiological studies, in our study, we reported similar findings by examining the association between genetically predicted blood lipids and LC. To our best knowledge, this is the first MR analysis to investigate the genetic association between common metabolic biomarkers and LC. Our findings not only provide complements to the previous results but also shed new light on the pathogenesis of LC.

To date, a few studies have reported a negative association between LDL and cancers. For instance, Alsheikh-Ali et al. reported an inverse association between on-treatment LDL levels and incident cancer in statin-treated patients enrolled in large randomized controlled trials [38]. A prospective cohort study also revealed that circulating levels of LDL may be negatively associated with the risk of cancers (hazard ratio < 1), albeit the association estimates were statistically non-significant [39]. A prospective cohort involved 68,759 Chinese male adults reported that circulating LDL levels was negatively associated with cancer risk (hazard ratio = 0.8) [40]. However, the association between blood LDL levels and LC risk has not been thoroughly investigated in population-based studies. In the current study, we found an inverse association between LDL and LC using MR approaches. The mechanisms underlying the negative association between LDL and LC are not yet well understood, although the biological roles of LDL in carcinogenesis have been proposed [41]. For example, low LDL has been proposed to be associated with suppressed immunity, upregulated activity or responsiveness of the mevalonate pathway, and increased activity of nuclear transcription factor NF-κB [42], thus promoting the initiation and progression of cancer. Metabolites of cholesterol, such as bile acid, have also been implicated in cancer progression [43]. However, the specific roles of LDL in lung tumorigenesis still need further investigations because LDL showed a distinct effect on the risk of different cancer sites, which may be driven by different mechanisms [43]. The observed genetic association between LDL and LC may also attribute to confounders that cause both a low plasma LDL cholesterol level and an increased risk of LC. One potential confounder is smoking, which could lead to low levels of LDL and increase the LC risk [44]. In the scenario of MR analysis, we could not completely tease out the confounding effect. Thus, concluding that LDL had a causal effect on LC should be cautious.

We also noted a significant association between genetically predicted TG and LC. In the univariate MR analysis, we found an inverse association between TG and LC. On the contrary, the MVMR analysis showed that genetically predicted TG was positively associated with LC. This finding is consistent between East Asians and Europeans and is in line with that of previous epidemiological studies [45, 46] and suggests that estimates of univariate MR analysis may be biased by other lipids. One possible mechanism relating TG to LC is that hypertriglyceridemia is associated with the development of oxidative stress and reactive oxygen species (ROS) [47]. On one hand, smoking is associated with elevated levels of TG [48], which may confound the association between TG and LC. However, Ulmer et al. found that the association remained when the data set was limited to non-smokers, suggesting that factors other than smoking status may contribute to the observed association [45].

We did not find a significant association between genetically predicted FPG and HbA1c and LC in both East Asians and Europeans, although the two diabetic factors have been shown to positively associate with LC risk in epidemiological studies [4, 49]. Our findings are consistent with previous MR studies. Yuan et al. reported a null association between genetically predicted diabetes and LC [50]. Torres et al. found that genetically predicted FPG was not significantly associated with LC, although there were only 24 SNPs that were used as the IVs [51]. These MR findings suggest that FPG and HbA1c may not be independent predictors for LC, rather than reflecting a risk status predisposing to LC.

A major strength of this study is the MR study design, which diminishes confounding and reverse causality potentially biasing the results in observational studies. We conducted our analyses on East Asians and Europeans. Thus, the results are easy to be extrapolated and compared between populations. Our study also has limitations. First, although there was no horizontal pleiotropy detected by MR-Egger regression, we could not conclude that LDL and TG were causally related to LC risk because we still cannot exclude that there is any direct causal pathway from the exposure-related genetic variants to cancer. Second, our MR estimates are not strictly consistent in East Asians and Europeans, suggesting that ethnic background may play a role in the examined association that deserves further investigation.

In conclusion, our MR study provides genetic evidence that blood LDL and TG are associated with LC in different directions among East Asians. However, these associations are not observed in Europeans. We did not detect a significant association between genetically predicted HDL, TC, FPG, and HbA1c and LC and its subtypes. The associations that reported in epidemiological studies may be driven by confounders or reverse causality.

## Supplementary Information

**Additional file 1: Table S1.** Genetic instrumental variables used for HDL in East Asians. **Table S2.** Genetic instrumental variables used for LDL in East Asians. **Table S3.** Genetic instrumental variables used for total cholesterol in East Asians. **Table S4.** Genetic instrumental variables used for triglyceride in East Asians. **Table S5.** Genetic instrumental variables used for fasting plasma glucose in East Asians. **Table S6.** Genetic instrumental variables used for HbA1c in East Asians. **Table S7.** Estimates of Mendelian randomization analyses on metabolic biomarkers and lung cancer in East Asians. **Table S8.** Genetic instrumental variables used for HDL in Europeans. **Table S9.** Genetic instrumental variables used for LDL in Europeans. **Table S10.** Genetic instrumental variables used for total cholesterol in Europeans. **Table S11.** Genetic instrumental variables used for triglyceride in Europeans. **Table S12.** Genetic instrumental variables used for fasting plasma glucose in Europeans. **Table S13.** Genetic instrumental variables used for HbA1c in Europeans. **Table S14.** Estimates of Mendelian randomization analyses on metabolic biomarkers and lung cancer in Europeans. **Table S15.** Estimates of multivariable Mendelian randomization analyses on metabolic biomarkers and lung cancer in Europeans.

**Additional file 2: Figure S1.** Estimates of Mendelian randomization analysis on metabolic biomarkers with LUAD. **Figure S2.** Estimates of Mendelian randomization analysis on metabolic biomarkers with LUSC. **Figure S3.** Estimates of Mendelian randomization analysis on metabolic biomarkers with SCLC. **Figure S4.** Scatter plot showing SNP effects on exposures and LUAD. A, fasting plasma glucose; B, HbA1c; C, HDL; D, LDL; E, total cholesterol; F, triglyceride. **Figure S5.** Scatter plot showing SNP effects on exposures and LUSC. A, fasting plasma glucose; B, HbA1c; C, HDL; D, LDL; E, total cholesterol; F, triglyceride. **Figure S6.** Scatter plot showing SNP effects on exposures and SCLC. A, fasting plasma glucose; B, HbA1c; C, HDL; D, LDL; E, total cholesterol; F, triglyceride. **Figure S7.** Mendelian randomization estimates using MRPRESSO method. A, East Asians; B, Europeans. **Figure S8.** Estimates of multivariable Mendelian randomization analysis on metabolic biomarkers with LUAD, LUSC, and SCLC.

## Data Availability

The GWAS summary data used in this study were available online and the corresponding websites are shown in Table 1.

## Abbreviations

FPG: Fasting plasma glucose
HbA1c: Glycosylated hemoglobin
HDL: High-density lipoprotein cholesterol
LDL: Low-density lipoprotein cholesterol
TC: Total cholesterol
MR: Mendelian randomization
LUCA: Lung cancer
LUSC: Lung squamous cell carcinoma
LUAD: Lung adenocarcinoma
SCLC: Small cell lung carcinoma
IVW: Inverse-variance weighted model
GWAS: Genome-wide association study
