# Supplementary material for: Genetic associations between circulating metabolic biomarkers and lung cancer in East Asians and Europeans
Source: Eur J Med Res. 2023 Apr 26;28:158. doi: 10.1186/s40001-023-01116-4 (PMC10131379; doi:10.1186/s40001-023-01116-4)

Figure S1. Estimates of Mendelian randomization analysis on metabolic biomarkers with LUAD.


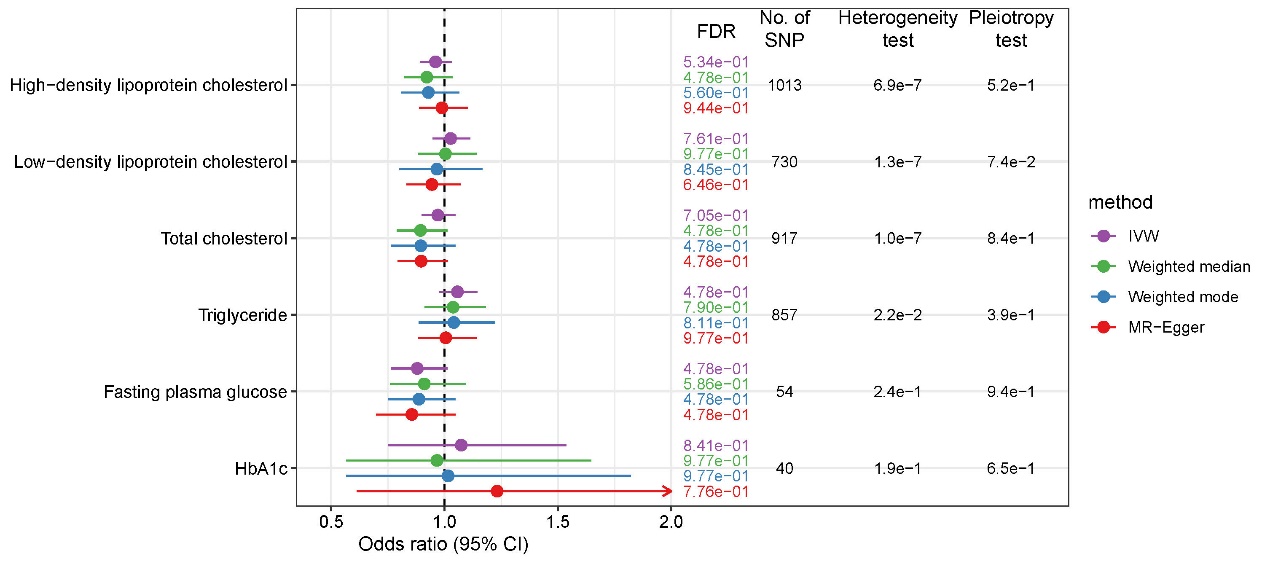


Figure S2. Estimates of Mendelian randomization analysis on metabolic biomarkers with LUSC.


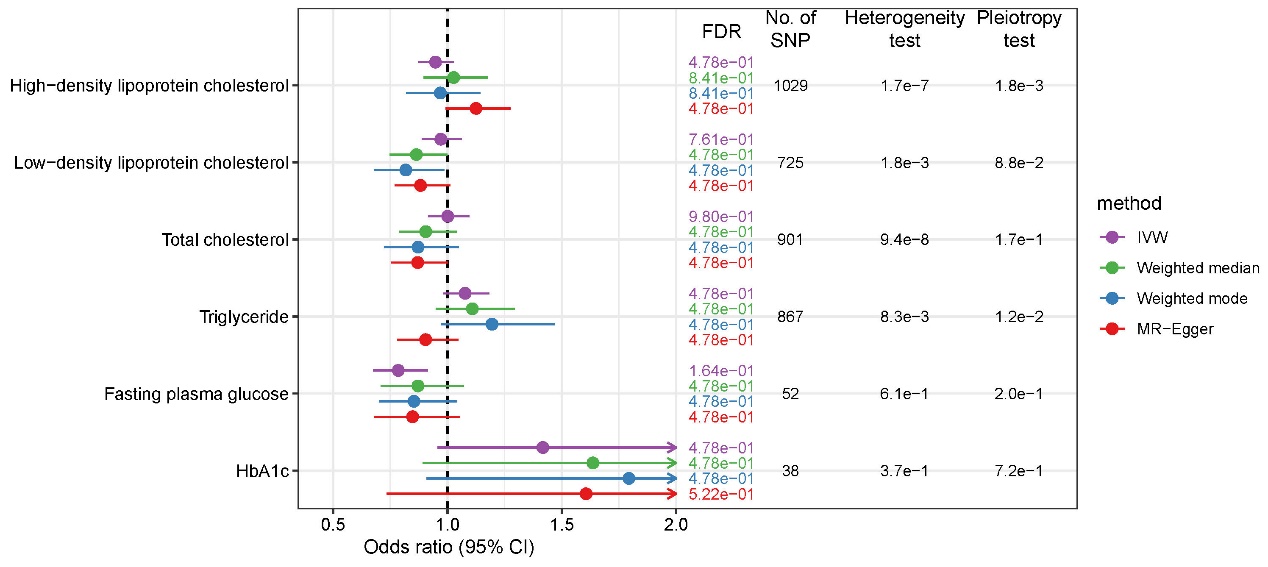


Figure S3. Estimates of Mendelian randomization analysis on metabolic biomarkers with SCLC.


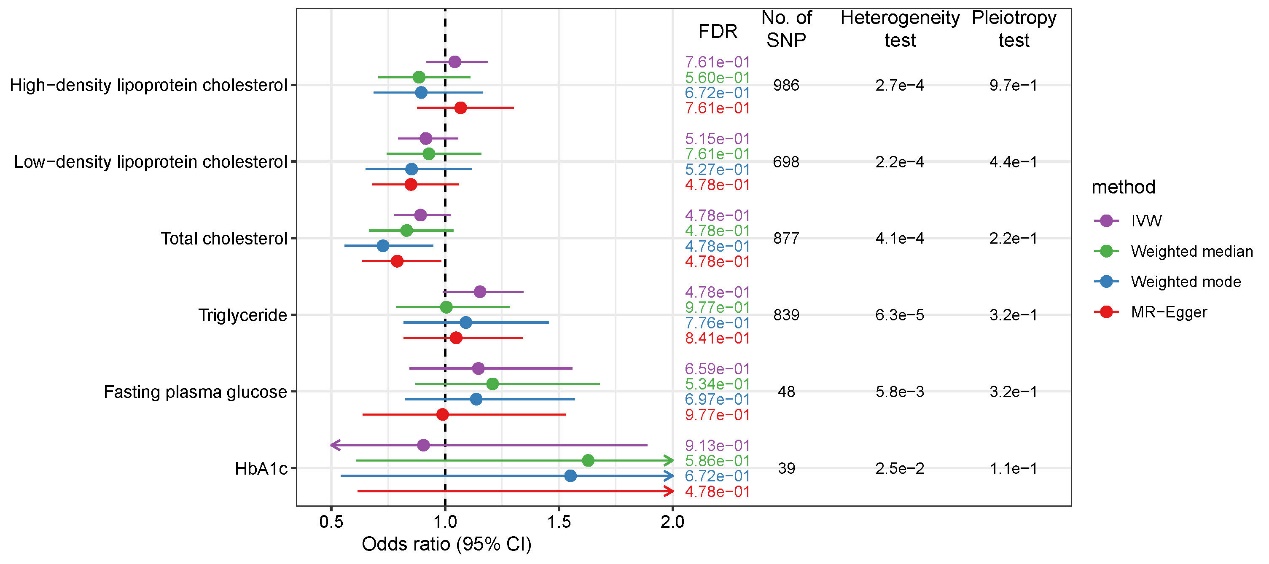


Figure S4. Scatter plot showing SNP effects on exposures and LUAD.

A, fasting plasma glucose; B, HbA1c; C HDL; D, LDL; E, total cholesterol; F, triglyceride.


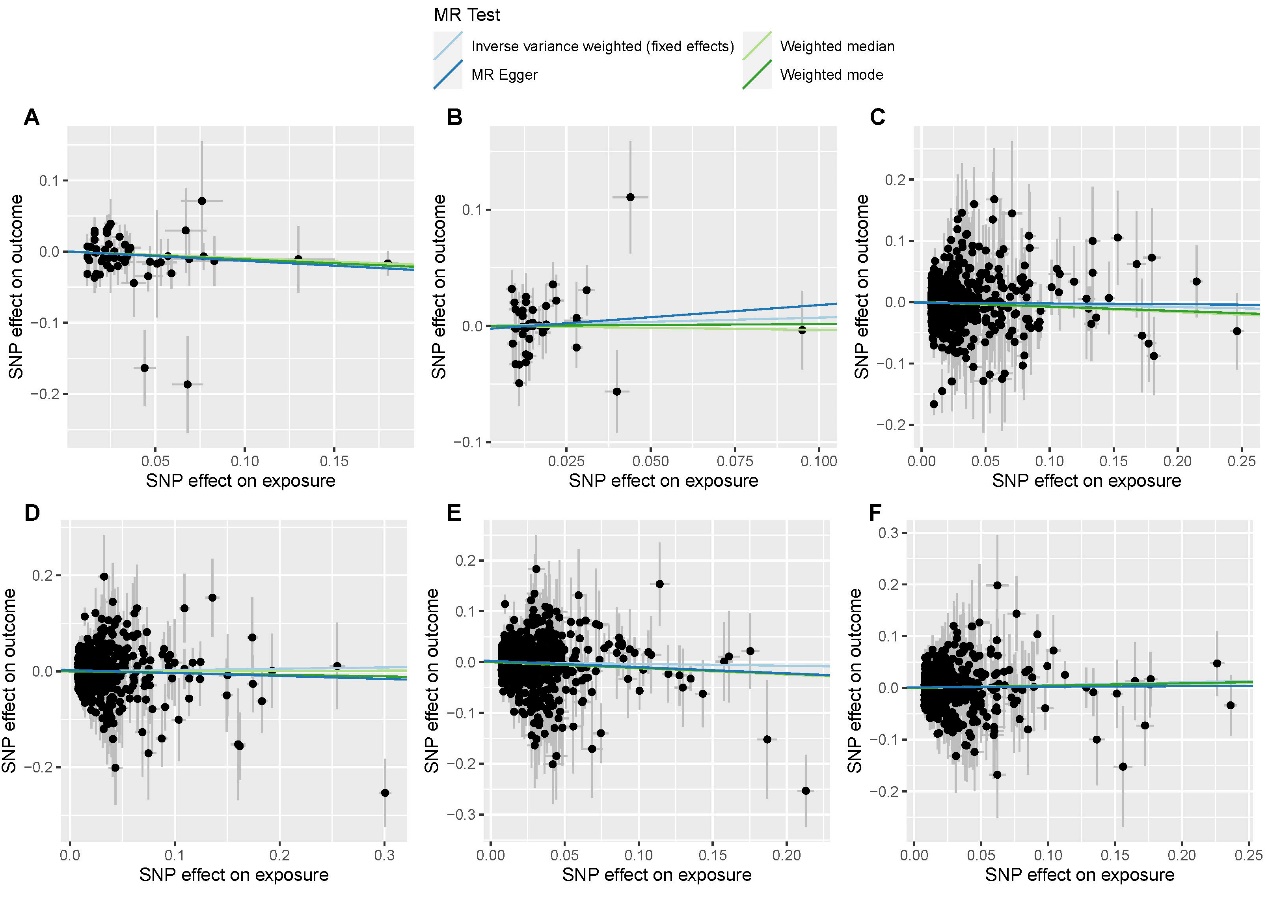


Figure S5. Scatter plot showing SNP effects on exposures and LUSC.

A, fasting plasma glucose; B, HbA1c; C HDL; D, LDL; E, total cholesterol; F, triglyceride.


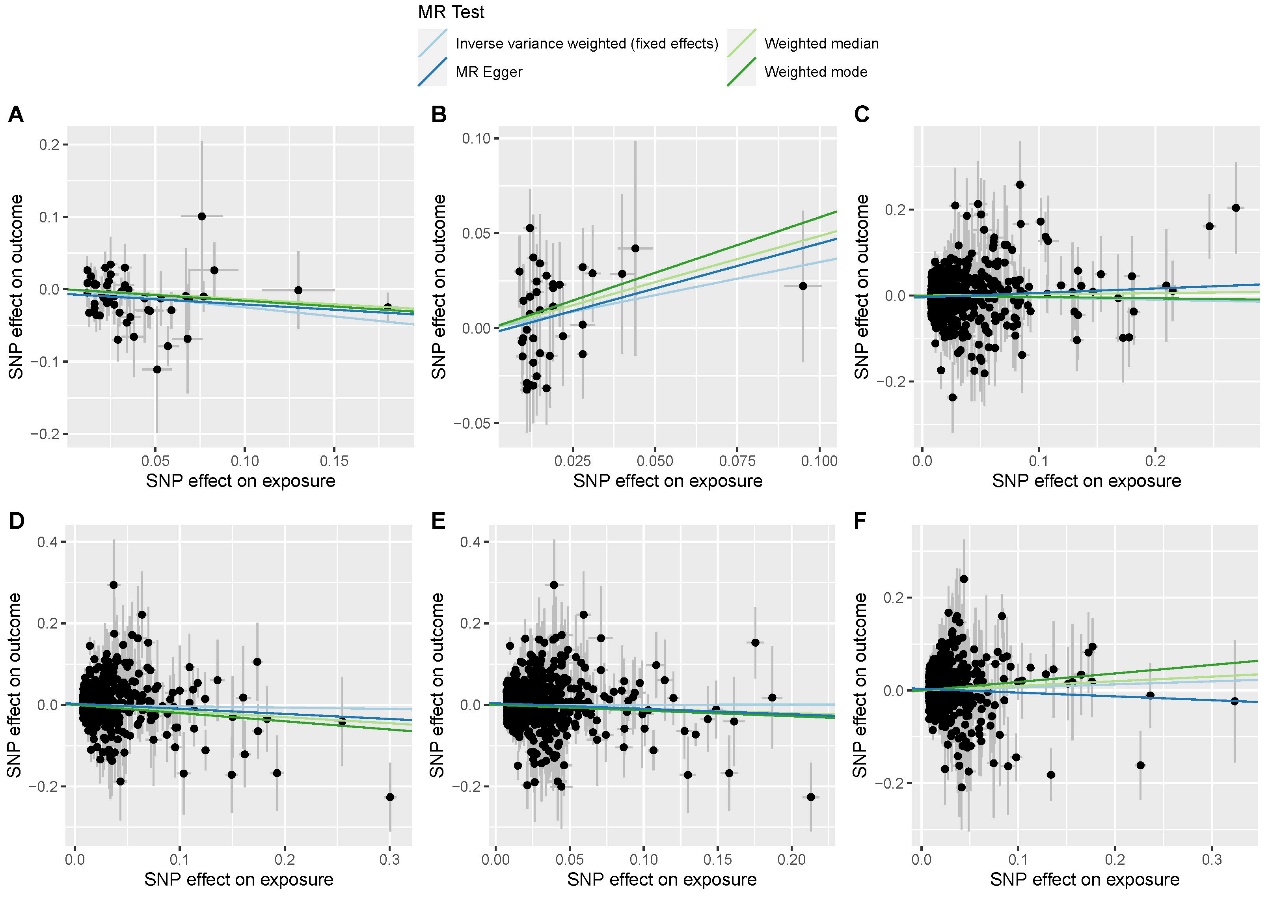


Figure S6. Scatter plot showing SNP effects on exposures and SCLC.

A, fasting plasma glucose; B, HbA1c; C HDL; D, LDL; E, total cholesterol; F, triglyceride.


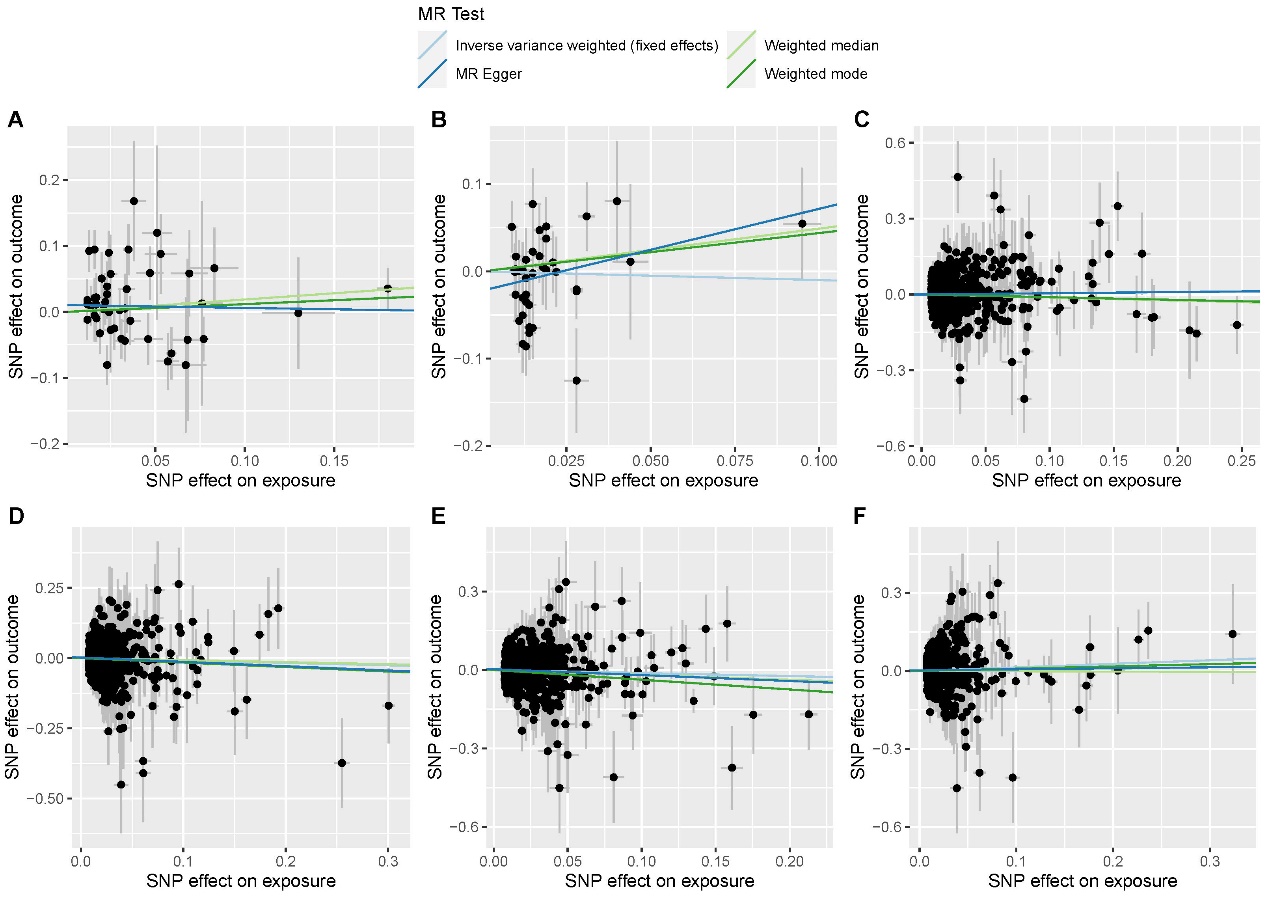


Figure S7. Mendelian randomization estimates using MRPRESSO method.

A, East Asians; B, Europeans.


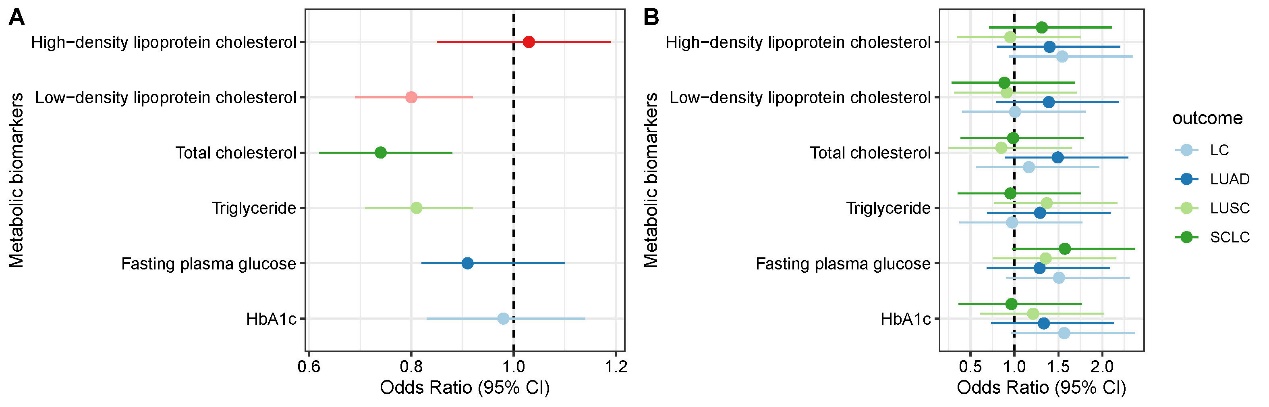


Figure S8. Estimates of multivariable Mendelian randomization analysis on metabolic biomarkers with LUAD, LUSC, and SCLC.


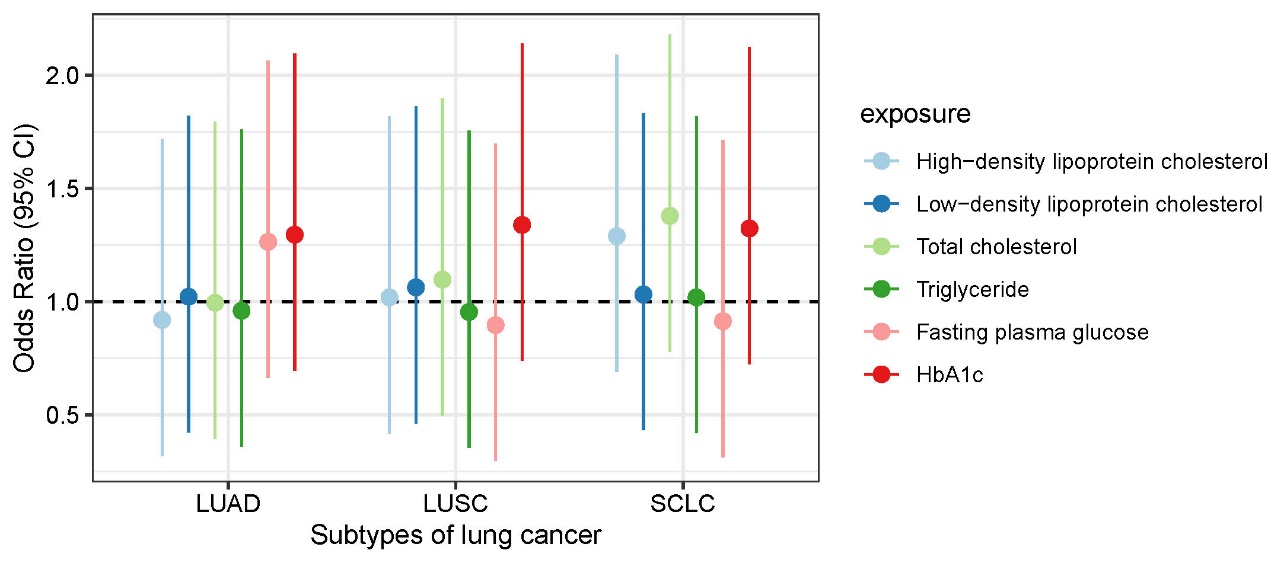

Supplement: Supplementary file 2 — Additional file 2: Figure S1. Estimates of Mendelian randomization analysis on metabolic biomarkers with LUAD. Figure S2. Estimates of Mendelian randomization analysis on metabolic biomarkers with LUSC. Figure S3. Estimates of Mendelian randomization analysis on metabolic biomarkers with SCLC. Figure S4. Scatter plot showing SNP effects on exposures and LUAD. A, fasting plasma glucose; B, HbA1c; C, HDL; D, LDL; E, total cholesterol; F, triglyceride. Figure S5. Scatter plot showing SNP effects on exposures and LUSC. A, fasting plasma glucose; B, HbA1c; C, HDL; D, LDL; E, total cholesterol; F, triglyceride. Figure S6. Scatter plot showing SNP effects on exposures and SCLC. A, fasting plasma glucose; B, HbA1c; C, HDL; D, LDL; E, total cholesterol; F, triglyceride. Figure S7. Mendelian randomization estimates using MRPRESSO method. A, East Asians; B, Europeans. Figure S8. Estimates of multivariable Mendelian randomization analysis on metabolic biomarkers with LUAD, LUSC, and SCLC. [file 40001_2023_1116_MOESM2_ESM.docx]
